# The effect of group-based cognitive behavioral therapy on inflammatory biomarkers in patients with coronary heart disease—results from the SUPRIM-trial

**DOI:** 10.1080/03009734.2018.1490829

**Published:** 2018-08-08

**Authors:** Erik M. G. Olsson, Fredrika Norlund, Ronnie Pingel, Gunilla Burell, Mats Gulliksson, Anders Larsson, Bo Karlsson, Kurt Svärdsudd, Claes Held

**Affiliations:** a Department of Women’s and Children’s Health, Uppsala University, Uppsala, Sweden;; b Department of Public Health and Caring Sciences, Uppsala University, Uppsala, Sweden;; c Department of Medical Sciences, Biomedical Structure and Function, Uppsala University, Uppsala, Sweden;; d Department of Medical Sciences, Cardiology, Uppsala University, Uppsala, Sweden

**Keywords:** Biomarkers, CBT, CHD, inflammation, stress management

## Abstract

**Background:** The Secondary Prevention in Uppsala Primary Healthcare Project (SUPRIM) is a prospective randomized controlled trial of a group-based cognitive behavioral therapy (CBT) stress management program for coronary heart disease (CHD) patients. The intervention reduced the risk of fatal or non-fatal first recurrent cardiovascular (CV) events. The aim of the present study was to analyze if the positive effects of the CBT program on clinical outcomes could have been mediated by changes in biomarkers for inflammation.

**Methods:** Altogether 362 patients with CHD were randomly assigned to intervention or usual care. The inflammatory biomarkers (VCAM-1, TNF-R1, TNF-R2, PTX3, and hs-CRP) were serially assessed at five time points every six months from study start until 24 months later, and analyzed with linear mixed models.

**Results:** Baseline levels of the inflammatory markers were near normal, indicating a stable phase. The group-based CBT stress management program did not significantly affect the levels of inflammatory biomarkers in patients with CHD. Three out of five (VCAM-1, TNF-R2, and PTX3) inflammatory biomarkers showed a slight increase over time in both study groups, and all were positively associated with age.

**Conclusion:** Group-based CBT stress management did not affect biomarkers for inflammation in patients with CHD. It is therefore unlikely that inflammatory processes including these biomarkers were mediating the effect the CBT program had on the reduction in CV events. The close to normal baseline levels of the biomarkers and the lack of elevated psychological distress symptoms indicate a possible floor effect which may have influenced the results.

## Introduction

Several psychological and stress-related risk factors for coronary heart disease (CHD) have been identified during the last decades (1). Negative emotional experiences and psychosocial stress increase the risk of both first and recurrent cardiac events. In the INTERHEART study, the combination of general and financial stress, stressful life events, depression, and low locus of control yielded a population attributable risk estimate of 33%. The independent effect of stress was consistent across geographic regions, in different age groups, and in both men and women (2). The array of psychological risk factors reflects both environmental triggers, the individual’s reaction to stressful burdens, and behavioral response patterns. Such factors are potentially behaviorally modifiable in preventive programs, for example through development of coping strategies, healthy behaviors, and problem-solving skills, and these programs are consequently suggested to reduce the risk of recurrent cardiac events (3). However, studies evaluating behaviorally oriented interventions targeting psychosocial risk factors, attempting also to influence cardiac outcomes and mortality, have shown inconsistent results. Successful interventions have often been group-based, behaviorally oriented, and focusing on stress management, while less successful interventions generally have been shorter and/or mainly individually based (4–6).

The Secondary Prevention in Uppsala Primary Health Care Project (SUPRIM) is a randomized controlled trial (RCT) studying patients with CHD to evaluate if a one-year group-based cognitive behavioral therapy (CBT) stress management program, in addition to usual care, would reduce cardiovascular (CV) outcomes compared with usual care only (7). The program in SUPRIM shared several characteristics with the successful interventions mentioned above. For example, it spanned over a year, was group-based, behaviorally oriented, and focused on stress management. The main finding during the 94 months of follow-up was a 41% reduction of fatal or non-fatal first recurrent CV events and a 45% reduction of recurrent myocardial infarction (MI). The outcome could not be explained by use of antihypertensives, lipid-lowering drugs, anti-depressants, or nicotine (7). Nor could it be explained by changes in stress behaviors, depression, or vital exhaustion (8). Only a minor positive effect of the program was found regarding somatic symptoms of anxiety. Thus, the mechanisms through which the program worked are still, to a large extent, unknown. However, several physiological and neuroimmunological mechanisms are potential candidates.

A bidirectional relationship between behavioral and emotional factors on one hand, and somatic systems, such as the CV and immune system, on the other, is well established (9). Depression, psychological distress, and job strain have been suggested to have a negative impact on inflammation and later CHD (10–13). When it comes to causality, depressive symptoms seem to predict inflammation in CHD patients rather than the other way around, although the relationship seems to be, to a large extent, mediated by health behaviors (14). Inflammation is thought to have a negative impact on CHD due to destabilization of atherosclerotic plaques and on myocardial contractility (13,15).

Only a few studies have reported on the dynamics of inflammatory biomarkers as outcomes of CBT-oriented interventions. In depressed subjects, with or without heart disease, CBT has been shown to have an effect on inflammatory biomarkers (16,17). Other studies, for example Claesson et al., who studied a CBT stress management program in women similar to the SUPRIM program, have not found any improvement in inflammatory biomarkers (18). More studies of CBT and its effects on biomarkers that may mediate disease progression are needed. In the present study we have focused on five selected inflammatory biomarkers previously used in CVD research, namely vascular cell adhesion molecule 1 (VCAM-1), tumor necrosis factor (TNF) receptor 1 (TNF-R1), TNF receptor 2 (TNF-R2), pentraxin 3 (PTX3), and high-sensitivity C-reactive protein (hs-CRP) (19–23).

The aim of the present study was to investigate if and to what extent inflammatory processes were affected by the CBT stress management program. If there was an association, the aim was also to investigate to what extent inflammatory processes could mediate the effect of the CBT program on CV events.

## Method

### Design and randomization

In the SUPRIM study CHD patients were randomized (1:1) to either a group-based CBT stress management program added to usual care or to usual care alone, which was the control condition. The main results have been previously presented (7). Measurements were collected from all participants five times every six months during 24 months. The CBT stress management program started between 0 and 11 months after randomization on a group-by-group basis.

### Study population

Patients who fulfilled the inclusion criteria (discharged from Uppsala University Hospital after an MI, a percutaneous coronary intervention [PCI], or a coronary artery bypass grafting [CABG] procedure, younger than 76 years, living in the hospital primary catchment area, Swedish speaking, and referred back to the general practitioner within 1 year from admission) were recruited between 1996 and 2002. Of the 812 patients initially assessed for eligibility 362 were included, of which 85 were women (23.5%), 185 (51.1%) were admitted for an MI, 122 (33.7%) for a CABG, and 55 (15.2%) for a PCI. The most commonly unmet inclusion criterion was the time criterion of being referred back to a general practitioner within a year (81%). This was mostly due to lacking hospital administrative routines. There were no differences in medical history or in risk factor measurements between the intervention group and the control group at baseline. Until the last follow-up, 94 months after randomization, 146 patients had at least one non-fatal CV event, 69 in the intervention group and 77 in the control group. In total, 48 patients died from CV- and non-CV-related causes during the full study period, 23 in the intervention group and 25 in the control group. For more details see (7).

The data in the present study were collected during the original SUPRIM study that was approved by the Regional Research Ethics Board (with registration numbers 2007/026, UPS 9658, and UPS03305).

### Procedure

Eligible patients were invited to participate after they had been referred back to their general practitioner. The hospital’s standard procedure was to do this approximately three months after discharge. Verbal informed consent was obtained according to the standard requirement at the time. A written invitation for a first (baseline) examination was then mailed to the patients, and those who accepted were eventually included. The procedure was repeated with new examinations including blood sampling at the 6th, 12th, 18th, and 24th month after baseline. Blood was drawn in the morning, after an overnight fast, from an antecubital vein with minimal occlusion, into evacuated glass tubes. Blood samples were frozen at –70°C, in serum and EDTA tubes, and stored in a freezer (7).

### The intervention

The one-year CBT stress management program contained five key components: education, self-monitoring, skills training, cognitive restructuring, and spiritual development, and focused on reducing daily experiences of stress such as time urgency and hostility. It was highly structured and followed a treatment manual. A detailed description of the intervention program has been provided elsewhere (24). Briefly, the program comprised 20 two-hour group sessions with 5 to 9 participants in gender-separated groups (25). The sessions were led by experienced psychologists, nurses, and a lay welfare worker who were all specially trained for this program. The therapists were supervised by the psychologist who designed the intervention (G.B., co-author of this paper). The same program has been used in other trials (25–27).

### Inflammatory biomarkers

The biomarkers used in the present study were selected among markers that have previously been shown to be associated with CV mortality or morbidity or kidney dysfunction in populations living in the same Swedish region/county as in the present study (20,28–30).

VCAM-1 mediates the adhesion of monocytes, lymphocytes, and other cells to the vascular endothelium, and reflects inflammation connected to early plaque development. It has been evaluated as a predictor of reduction of endothelial cell function and predictor of coronary heart disease (31,32).

TNF is a potent cytokine acting as a key regulator of the inflammatory response. It is produced by many cell types: macrophages, monocytes, T and B lymphocytes, keratinocytes, endothelial cells, osteoclasts, neutrophils, and fibroblasts (33–36). TNF binds to two different receptors (R). TNF-R1 is expressed in almost all cells in the body, and TNF-R2 is expressed on subtypes of neurons, oligodendrocytes, microglia, and astrocytes in the brain, on endothelial cells, CD4 and CD8 T cells, cardiac monocytes, thymocytes, and mesenchymal cells (37). TNF-R1 activates signaling pathways leading to cell death. TNF-R2 can activate numerous changes in the gene expression that drives inflammation, cell proliferation, and cell survival (38,39). TNF also plays a role in a number of CV diseases (40,41), and anti-TNF therapy to patients with rheumatoid arthritis reduces the risk for CV events (42).

PTX3 is produced mainly in atherosclerotic plaques, dendritic cells, neutrophils, macrophages, smooth muscle cells, and endothelial cells. It has been studied as a marker of low-grade inflammation (30,43).

High-sensitivity CRP is an acute phase protein synthesized in the liver following interleukin-6 secretion and is extensively used as a downstream inflammatory biomarker in atherosclerotic disease. However, its mechanistic role has been questioned (44).

VCAM, TNF-R1, TNF-R2, and PTX3 were analyzed by commercial sandwich ELISA kits, (DY809, DY225, DY726, and DY1826; R&D Systems, Minneapolis, MN, USA). The total coefficient of variations of the assays were approximately 6%. High-sensitivity CRP was analyzed on a BS380 instrument (Mindray, Shenzhen, China) with reagents from Abbott Laboratories, (6K26; Abbott Park, IL, USA). The total coefficient of variation for the CRP method was 6.9% at 1.30 mg/L. All assays were performed blinded without knowledge of the study groups. For expected values from healthy individuals for all five biomarkers, see the note in Table 1.

**Table 1.** Baseline characteristics of the study population.

	Intervention (*n* = 192)	Control (*n* = 170)
Age at baseline, mean (SD), y	62.0 (7.94)	61.0 (8.28)
Sex, *n* (%) females	43 (22.4)	42 (24.7)
Marital status, *n* (%) married	150 (78.1)	132 (77.6)
Highest educational level, *n* (%)	(*n* = 189)	(*n* = 161)
Compulsory education	67 (35.4)	62 (38.5)
Vocational training	62 (32.8)	57 (35.4)
High school	22 (11.6)	10 (6.2)
University or college education	38 (20.1)	32 (19.9)
Disability pensioner, *n* (%)	33 (17.2)	15 (8.8)
Old-age pensioner, *n* (%)	96 (50.0)	76 (44.7)
Heart failure, *n* (%)	45 (24.6)	42 (26.8)
Previous myocardial infarctions, *n* (%)	60 (31.3)	60 (35.3)
VCAM-1, mean (SD), µg/L	374 (112)	375 (119)
TNF-R1, mean (SD), µg/L	1.51 (0.37)	1.57 (0.52)
TNF-R2, mean (SD), µg/L	3.79 (0.96)	3.86 (1.31)
PTX3, mean (SD), µg/L	2.09 (1.09)	2.39 (2.09)
hs-CRP, mean (SD), mg/L	4.29 (5.8)	4.14 (4.29)

Expected values when analyzing samples from healthy individuals according to the manufacturer of the respective assays are: VCAM-1 < 900 µg/L, TNF-R1 < 1.97 µg/L, TNF-R2 < 3.17 µg/L, PTX3 < 1.18 µg/L, hs-CRP <5 mg/L.

### Statistical analyses

All results were based on the assigned treatment at randomization (intention-to-treat). To study the effect of the intervention, the interaction term between groups and time in the fixed effect part of linear mixed models (LMM) was tested. The interaction term describes whether the patients in the two groups had different mean trajectories across the observation period. The baseline measurement was retained as a part of the outcome. Because the intervention was randomized, the intervention and control group were assumed to have equal background characteristics at baseline. To account for the repeated measures design, LMMs include random effects based on likelihood ratio tests. Restricted maximum likelihood was used to estimate the models. Maximum likelihood estimation is efficient since it uses all available observations and is independent of the dropout under the missing at random assumption (45). To improve the efficiency, adjusted regression models that included sex, age, education, and previous MI were done in addition to the crude models. Education was dichotomized to university level or not. Residual analyses and a check for outliers were performed to assess model adequacy. Logarithmic transformations of all five outcomes were carried out accordingly and were thereafter used in the main analyses. A *post hoc* power calculation shows that the sample size (*n* = 362) is large enough (80% power, α = 0.05) for detecting a Cohen’s *d* effect size of 0.3 (the difference in means divided by the pooled standard deviation), which is considered being between small and moderate. Additional analyses were conducted including only patients with higher-than-median baseline levels of each inflammatory biomarker. These analyses replicated the main analyses and included 175 patients. Data on biomarkers were missing for between 6 and 18 (3%–9%) participants in the intervention group and between 10 and 21 (6%–12%) in the control group at the different time points. All analyses were performed in R 3.1.1 using the package nlme (46,47).

## Results

The two study groups were well balanced in terms of baseline characteristics. There were no differences between the intervention group and the control group regarding age, sex, marital status, educational level, retirement, or any of the psychological outcomes (Table 1).

**Figure 1.**
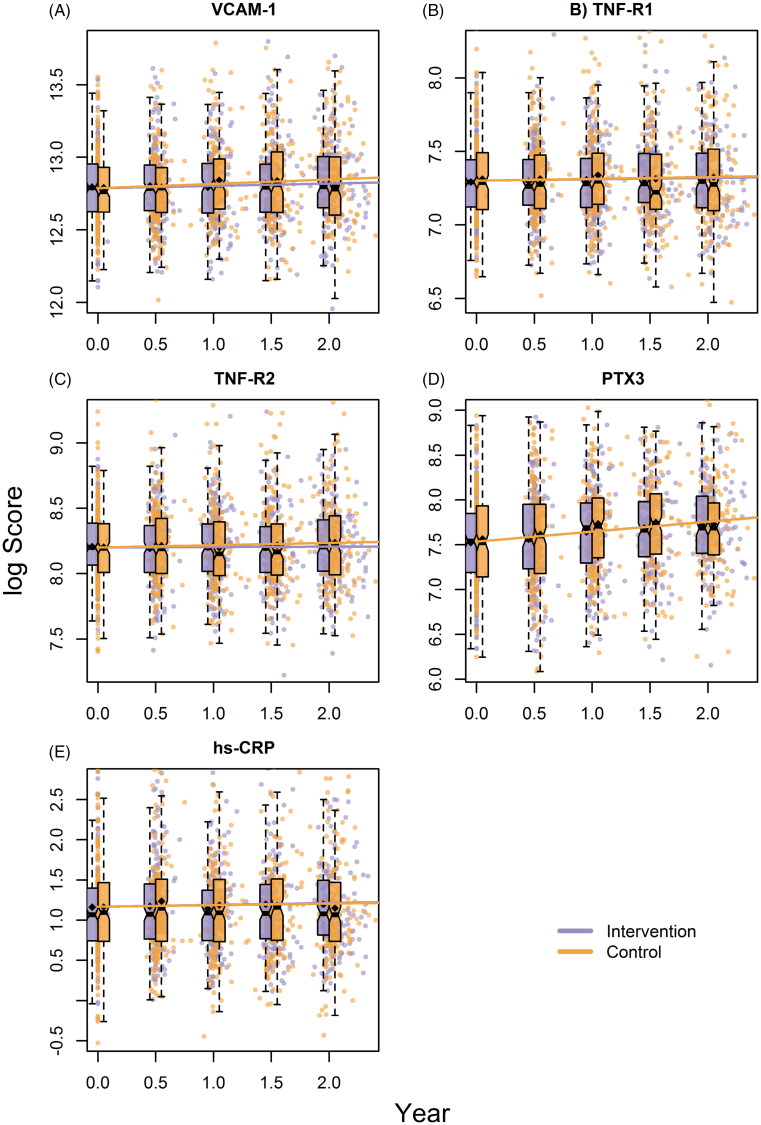
Change over time of the five inflammatory biomarkers by randomized treatment. The change is shown with boxplots and estimated group means (shown as ?) together with fitted lines from the crude linear mixed models in Table 2.

The baseline levels of the biomarkers were near normal on average compared to healthy reference values (Table 1). No effect of the CBT stress management program on levels of any of the inflammatory biomarkers over time was found (Table 2; Figure 1). The results did not change when adjustments for age, sex, education, and previous MIs were performed. VCAM-1 and PTX3 increased over time in both groups in both regression models. TNF-R2 also showed an increase over time in both groups but with less precision. All biomarkers showed a positive relationship with age, and none was associated with sex (Table 2). Additional replications of the analyses including only patients showing higher-than-median baseline levels of the separate inflammatory biomarkers were conducted. These analyses confirmed that there were no treatment effects even for patients with higher baseline levels of inflammatory biomarkers (crude estimates of the Group × Time interaction: VCAM-1 = –0.03, *P* = 0.17; TNF-R1 = –0.02, *P* = 0.18; TNF-R2 = –0.03, *P* = 0.08; PTX3 = –0.01, *P* = 0.65; hs-CRP = –0.05, *p* = 0.30). The adjusted effects were very similar.

**Table 2.** Estimated fixed effects in inflammatory biomarkers, crude and adjusted model.

	VCAM-1		TNF-R1		TNF-R2		PTX3		hs-CRP	
	Crude (95% CI)	Adjusted (95% CI)	Crude (95% CI)	Adjusted (95% CI)	Crude (95% CI)	Adjusted (95% CI)	Crude (95% CI)	Adjusted (95% CI)	Crude (95% CI)	Adjusted (95% CI)
Group*Time	−0.01	−0.02	−0.003	−0.01	−0.01	−0.02	0.001	−0.004	0.004	0.001
	(−0.04, 0.01)	(−0.04, 0.01)	(−0.02, 0.02)	(−0.03, 0.01)	(−0.04, 0.01)	(−0.04, 0.01)	(−0.04, 0.05)	(−0.05, 0.04)	(−0.05, 0.06)	(−0.06, 0.06)
Time (year)	0.03**	0.03**	0.01	0.01	0.02	0.02*	0.11***	0.11***	0.02	0.02
	(0.01, 0.05)	(0.01, 0.05)	(−0.003, 0.03)	(−0.002, 0.03)	(−0.0001, 0.04)	(0.001, 0.04)	(0.07, 0.15)	(0.08, 0.15)	(−0.03, 0.06)	(−0.02, 0.06)
Age (year)		0.01***		0.02***		0.02***		0.01***		0.01***
		(0.01, 0.01)		(0.01, 0.02)		(0.01, 0.02)		(0.01, 0.02)		(0.01, 0.02)
Sex (male)		−0.02		−0.03		−0.03		0.06		−0.11
		(−0.07, 0.03)		(−0.09, 0.02)		(−0.09, 0.03)		(−0.05, 0.17)		(−0.25, 0.03)
Education (university)		−0.04		0.02		−0.01		0.18**		0.01
		(−0.09, 0.02)		(−0.04, 0.08)		(−0.07, 0.06)		(0.06, 0.29)		(−0.14, 0.15)
PMI		−0.01		−0.002		−0.001		−0.01		−0.08*
		(−0.03, 0.02)		(−0.03, 0.03)		(−0.03, 0.03)		(−0.07, 0.05)		(−0.15, −0.001)
Constant	12.8***	12.3***	7.30***	6.37***	8.20***	7.29***	7.53***	6.65***	1.17***	0.45
	(12.8, 12.8)	(12.1, 12.5)	(7.27, 7.33)	(6.17, 6.56)	(8.17, 8.23)	(7.08, 7.50)	(7.48, 7.59)	(6.26, 7.04)	(1.10, 1.23)	(−0.04, 0.95)
Observations	1664	1664	1665	1665	1665	1665	1665	1665	1665	1665

Logarithmized values of the biomarkers were used in the analyses.

*P < 0.05; **P < 0.01; ***P < 0.001.

CI: confidence interval; PMI: previous myocardial infarction.

Since no effect of the intervention on the biomarkers was found, no analyses of mediations were done.

## Discussion

Although the SUPRIM study has shown a positive treatment effect in terms of reducing recurrent CV events and MIs, the present results showed no effect of the CBT program on any of the five inflammatory biomarkers tested. Inflammatory processes are therefore unlikely as mediators between the CBT program and the positive results previously reported (7). It has also been shown that other measures—such as psychological outcomes, social support, physical activity, blood pressure, blood lipid levels, smoking, and use of secondary preventive medications or anti-depressants—were likewise unaffected by the treatment and therefore unlikely to explain the reduction in CV events and MIs (8). In order to explain the SUPRIM study’s main finding, the most important mechanisms are still to be revealed. If the mediating mechanisms were at all included among the study’s variables they must either have not been captured adequately, or else several diverse smaller changes may have worked synergistically to make a difference, for example attention from the group, more visits to the clinic, subtle life-style changes, or improved health literacy.

In three out of the five biomarkers at least one of the models indicated an effect of time, and all were associated with age. These results might potentially be an effect of slightly worsened kidney function with time and with age, together with a possible slow progression of atherosclerosis. The selection of biomarkers was made based on evidence from previous studies, but we cannot rule out that other markers of inflammation might have been affected by the treatment, although this is unlikely considering the usually high correlation between different inflammatory biomarkers and the almost zero-effect in the present study.

Inflammation has been proposed as a mediating variable between behavioral and emotional risk factors and cardiac events. However, most of the studies that support such a mechanism have included patients suffering from depression (14). This goes also for studies that showed a positive effect of a CBT-oriented treatment (16). In the present study the patients did not report higher levels of depression or any other psychological distress than a matched non-CHD control group (48). In line with this, the baseline levels of most biomarkers were also close to normal considering the patients’ age. This indicates that patients were recruited in a stable phase and not too close to any acute event, which is especially important to note since some patients had gone through a CABG. However, it is also a challenge as it implicates the existence of a floor effect which prevents the detection of improvements. But since there are typically deteriorations over time and with age in these measures, which was also shown in this study, it would still have been possible to detect protective effects. The results did not change much when excluding the 50% of patients with the lowest baseline levels.

A methodological limitation in this study was that the assessments were timed with the inclusion into the study and not with the onset of the treatment, which sometimes was delayed up to a year after the first assessment. This makes it more difficult to detect true intervention-related changes in biomarkers. A second limitation was that participants in the study were not recruited based on an assessment of the need of this particular intervention but rather on their discharge diagnosis. This may have led to less motivated participants. However, it does not explain the difference in results between CV events and inflammatory biomarkers.

In conclusion, a group-based CBT stress management program in patients with CHD which showed a beneficial effect in reducing long-term CV events seems not to have had any effect on biomarkers reflecting inflammatory processes. There is still a need to better understand the mechanisms in successful stress management programs in clinical and research settings.
